# Morphology and root canal configuration of maxillary canines: a systematic review and meta-analysis

**DOI:** 10.1186/s12903-024-04682-z

**Published:** 2024-08-15

**Authors:** Thomas Gerhard Wolf, Theodora Rempapi, Richard Johannes Wierichs, Andrea Lisa Waber

**Affiliations:** 1 Department of Periodontology and Operative Dentistry, University Medical Center of the Johannes Gutenberg-University Mainz, Mainz, Germany; 2 Department of Restorative, Preventive and Pediatric Dentistry, School of Dental Medicine, University of Bern, Bern, Switzerland

**Keywords:** Internal morphology, Maxillary canine, Meta-analysis, Root canal configuration, Systematic review

## Abstract

**Background:**

This study assessed the internal morphology of maxillary canines (MxC) through a systematic review of existing literature.

**Methods:**

Research articles up to June 2024 were retrieved from five electronic databases (MEDLINE via PubMed, Embase, Scopus, LILACS, and Cochrane). Predefined search terms and keywords were used, and potential studies were identified by cross-referencing and bibliographies of the selected articles reviewed.

**Results:**

Two hundred studies were identified, 73 duplicates were removed, 127 records were screened, and 113 were removed after consultation of title and abstract. After full-text consultation and hand searching, finally 22 studies were included. Using the method for describing the root canal configuration (RCC) of Briseño Marroquín et al. (2015) and Vertucci (Ve) (1984), the most frequently reported RCC of MxC were 1–1-1/1 (Ve I, 75.4–100%), 2–2-1/1 (Ve II, 0.1–20%), 1–2-1/1 (Ve III, 0.1–11.6%), 2–2-2/2 (Ve IV, 0.1–0.4%), 1–1-2/2 (Ve V, 0.1–2.4%), 2–1-2/2 (Ve VI, 0.5–1.2%), and 1–2-1/2 (Ve VII, 0.1–0.2%). The meta-analysis of six studies (Europe/Asia) showed that a significantly higher number of RCC of 2–2-1/1 (Ve II) (OR [95%CI] = 1.34 [0.53, 3.41]), 1–2-1/1 (Ve III) (OR [95%CI] = 2.07 [1.01, 4.26]), and 1–1-2/2 (Ve V) (OR [95%CI] = 2.93 [1.07, 8.07]), were observed in males, and 2–2-2/2 (Ve IV) (OR [95%CI] = 0.08 [0.00, 4.00]) in females. No sex differences in the RCC of 1–1-1/1 (Ve I) and 1–2-1/2 (Ve VII) were observed.

**Conclusions:**

Cone beam computed tomography is the most frequently used method for research on the RCC of MxC. Despite the high prevalence of type 1–1-1/1 (Ve I) RCC in MxC, clinicians should remain vigilant for more complex and sex-differentiated patterns in up to 25% of cases to prevent endodontic treatment complications or failures.

**Supplementary Information:**

The online version contains supplementary material available at 10.1186/s12903-024-04682-z.

## Introduction

Detailed knowledge and comprehensive understanding of the three-dimensional internal morphology and root canal configuration are crucial for the success of endodontic treatment [1–3]. Awareness of the complexity of root canal anatomy simplifies the planning of endodontic therapy and the respective treatment steps, furthermore, diminishes the possibility of iatrogenic errors [1–4]. The existence of numerous morphological differences emphasizes the importance of diagnosing and evaluating each case individually. Numerous studies show that the most frequent root canal configuration (RCC) of single-rooted maxillary canines is a single root canal from the pulp chamber to the apex (1–1-1/1, Vertucci I) [5–9]. However, different populations studied using modern 3D imaging examination methods show that up to a quarter of the teeth have anatomical variations [7–13], which can offer problems and additional challenges for the different steps of root canal treatment that could lead to failure. In the past, various ex vivo methods have been used to study the morphology of root canal systems such as clearing technique, scanning electron microscopy, and light microscopy [1, 2, 5, 6, 14, 15]. Cone beam computed tomography (CBCT) has proven to be a modern and particularly effective tool for such in vivo examinations, as it offers a superior level of detail compared to previous methods [16, 17], even if CBCT is inferior to micro-computed tomography regarding imaging of fine structures and details [18]. The combination of three-dimensional imaging and software analysis allows a non-destructive clinical examination of complex internal morphological structures of the root canal system without compromising the integrity of the tooth [16, 17]. The classifications of RCC proposed by Vertucci [1] and Weine et al. [2] et al. describe possible root canal system variations; unfortunately, they cannot respond to the morphological intricacies of some root canals in comparison to more modern classifications describing root canal anatomy or root canal configuration [3, 4]. The current study aimed to systematically review the literature on the internal morphology and in particular root canal configuration of maxillary canines (MxC) and to identify sex influence on variation in root canal morphology.

## Materials and methods

A systematic review was undertaken with the aim to examine the published literature on the internal morphology and root canal configuration (RCC) of MxC up to June 2024. The following five databases were searched: MEDLINE via PubMed, Embase, Cochrane Database, LILACS, and Scopus as well as grey literature. The Preferred Reporting Items for Systematic Reviews and Meta-Analyses (PRISMA) guidelines were followed by the current systematic review [19]. Furthermore, the review protocol was registered in the International Prospective Register of Systematic Reviews (PROSPERO) system (CRD42023394478). In the review protocol inclusion criteria were defined as follows: randomized controlled trials, cross-sectional studies, comparative, validation, and evaluation studies of the internal morphology and RCC of MxC without any restrictions. Case reports and reviews were excluded. A standardized comprehensive search strategy was used, including a combination of MeSH terms and keywords: (“root canal configuration” OR “root canal system” OR “root canal morphology”) AND (“maxillary canine” OR “maxillary anterior teeth”) AND (“morphology” OR “anatomy”). Cross-referencing and hand-search were performed by using the bibliographies of full-text articles. Studies addressing morphological anatomies other than the internal morphology and root canal configuration of maxillary canines were excluded. Duplicates or repeated articles were removed, and the remaining ones were evaluated based on their title and abstract by two independent reviewers (T.R., A.L.W.). Papers not relevant to the topic were discarded again at this stage. The remaining papers underwent a full-text review and were examined again by the same two independent reviewers. All the included articles were summarized in a table, referring to the following details: authors, publication year, quality assessment, place of origin, number of samples, methodology, sex (if mentioned), and root canal configuration using the classifications of Vertucci [1], Weine et al. [2], and Briseño Marroquín et al. [3]. The risk of bias was evaluated using the Anatomical QUality Assessment (AQUA) tool [20], specifically designed for assessing the quality of anatomical studies included in meta-analyses and systematic reviews. Two independent reviewers (T.R., A.L.W.) screened the articles for bias assessment. In case of disagreement, a third reviewer (T.G.W.) was consulted to achieve consensus. The quality of the included studies was assessed by two independent reviewers (A.L.W., T.G.W.) following the customized quality assessment tool developed by the National Heart, Lung, and Blood Institute (www.nhlbi.nih.gov/health-topics/study-quality-assessment-tools).

The statistical analysis of the included studies for the meta-analyses was performed using Review Manager software (RevMan version 5.4, Cochrane Collaboration, Copenhagen, Denmark, 2014). The odds ratio (OR) was used to determine the effect size. The I^2^ statistic was used to quantify the degree of variability between studies, which was due to heterogeneity rather than chance [21]. Based on the degree of heterogeneity (I2 < 35% for low heterogeneity, fixed-effects meta-analysis; I^2^ > 35% for substantial heterogeneity, random-effects meta-analysis), the appropriate meta-analysis model was selected [22, 23]. The primary outcome measures comparing different root canal configurations, patient sex, and geographical factors were presented as odds ratios with 95% confidence intervals (95% CI) for studies with binary outcomes. A *p*-value of 0.05 or less was considered statistically significant.

## Results

**Fig. 1:**
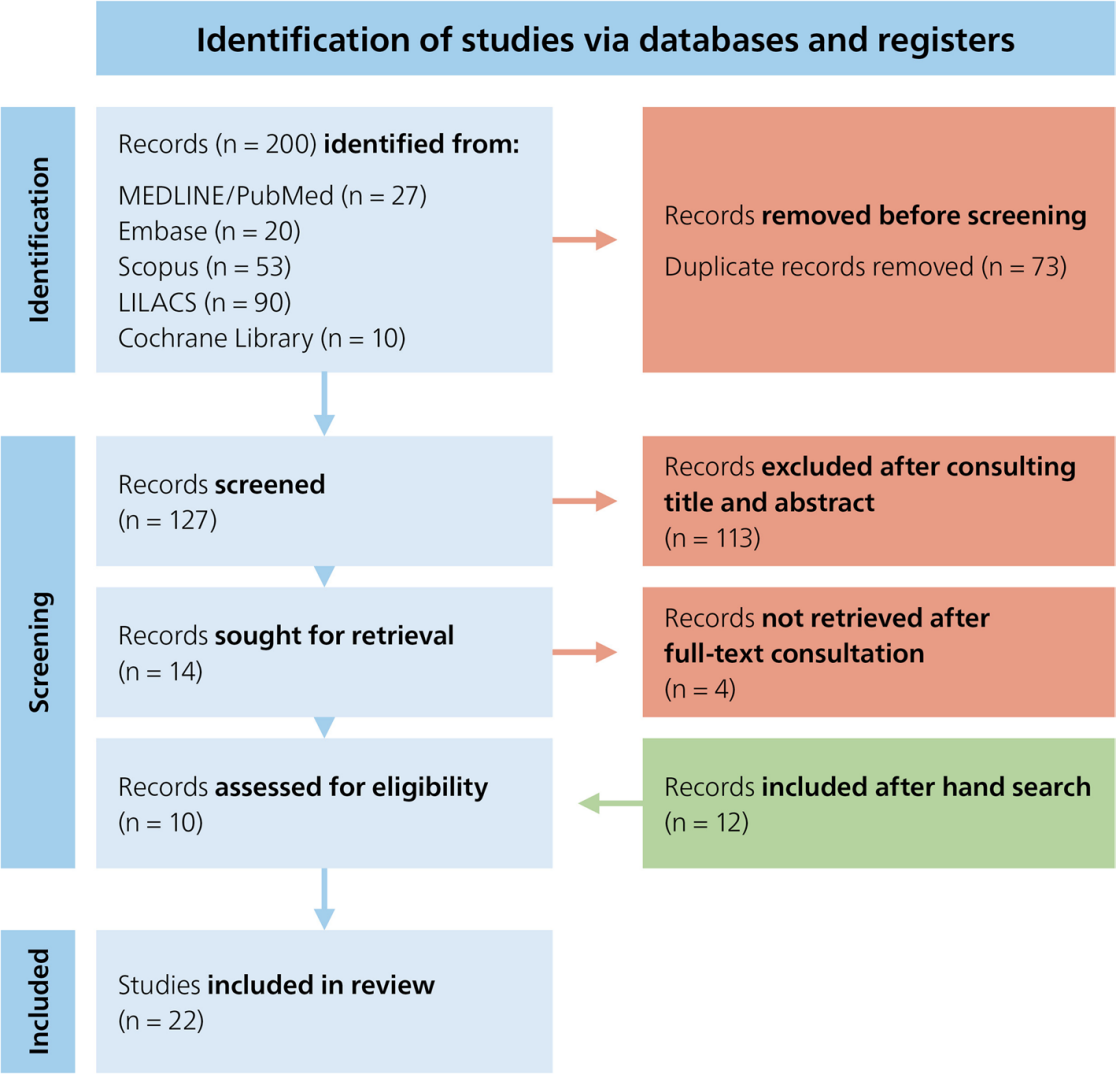
PRISMA flow diagram

The literature search through five databases resulted in 200 articles. The 127 remaining articles after removing all duplicates were screened by title and abstract. 14 articles were consulted in full text and four articles were excluded. Twelve articles were added to this investigation after a hand search, resulting in a total of 22 reviewed articles. The selection process is shown in a PRISMA flowchart diagram [19] (Fig. 1). Data of the risk of bias assessment using the Anatomical QUality Assessment (AQUA) tool can be found in Supplementary Materials. The included investigations were conducted in various regions and populations around the world, utilizing different methodologies, encompassing both sexes and without age limitations.

**Fig. 2:**
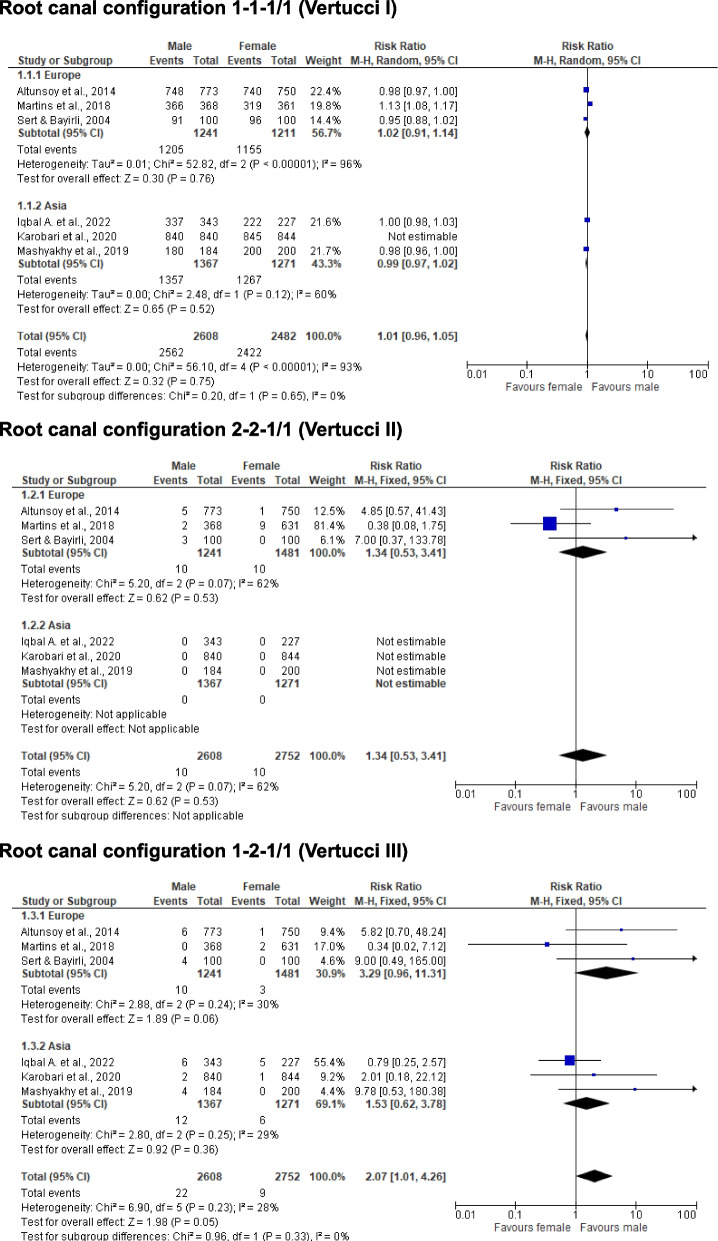
Root canal configuration 1–1-1/1 (Vertucci I), 2–2-1/1 (Vertucci II), and 1–2-1/1 (Vertucci III)

**Fig. 3:**
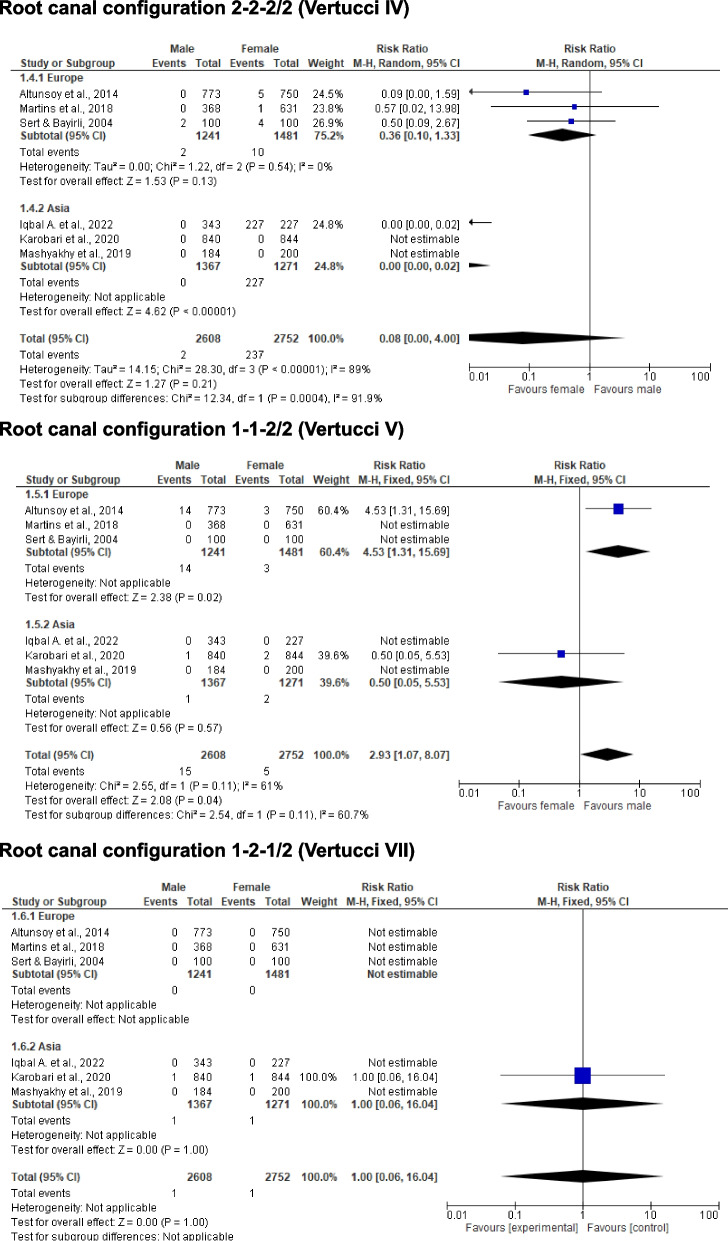
Root canal configuration 2–2-2/2 (Vertucci IV), 1–1-2/2 (Vertucci V), and -2–1/2 (Vertucci VII)

Table 1 shows a summary of the included studies regarding the RCC of MxC until December 2023. The table is divided into detailed information on the author(s), publication year, quality assessment, population, sample number, research method, and RCC according to the classification proposed by Vertucci [1], and Weine et al. [2], and Briseño Marroquín et al. [3]. The most frequently observed RCC of MxC, regardless of the research methodology or the sample number, is 1–1-1/1 (Vertucci’s I or Weine et al. I) with a frequency of 75.4 – 100% [1, 5, 7–15, 24–33]. The second most common RCC in MxC is Briseño Marroquín et al.’s 2–2-1/1 (also known as Vertucci’s II; Weine’s II) with 0.1–20% [12, 15, 23–25, 27, 28], and the following common RCC with frequency up to 11.6% is Briseño Marroquín et al.’s type 1–2-1/1 (Vertucci’s III) [6, 7, 9, 11, 12, 14, 15, 24–29, 32, 33], whereas the Weine et al. classification does not include this RCC type. Among the summarized studies in Table 1, other RCCs such as 2–2-2/2 (Vertucci IV or Weine et al. III), 1–1-2/2 (Vertucci V), 2–1-2/2 (Vertucci VI) and 1–2-1/2 have been also observed less frequently. The CBCT analysis is reported as the most used research method [7–13, 25, 26, 28–33], with the radiographic [5], staining and clearing [1, 14, 15, 24], or micro-computed tomography [27] methods less frequently reported. Six studies reported sex-specific differences [8, 11, 13, 28, 29, 32]; data from the meta-analysis of these studies differed by origin (Europe/Asia) are shown in Figs. 2 and 3.

**Table 1:** Included studies summarizing various comparative and non-comparative morphological studies of the root canal configuration (RCC) of maxillary canines (MxC). The RCC are described according to the classifications of Vertucci (Ve) [1], Weine et al. (We) [2], and Briseño Marroquín et al. (Br) [3]

Report		PP	n	Met	RCC-frequency (%)								
Authors, Year	Q/R	RCC		Ve (1984)	I	II	III	IV	V	VI	VII	VIII	^a^
				We (1969)	I	II		III					^a^
				Br (2015)	1–1-1/1	2–2-1/1	1–2-1/1	2–2-2/2	1–1-2/2	2–1-2/2	1–2-1/2	1–1-3/3	^a^
Pineda & Kuttler, 1972 [5]	G	MEX	260	Rx	100.0	-	-	-	-	-	-	-	-
Vertucci F., 1984 [1]	G	USA	100	SC	100.0	-	-	-	-	-	-	-	-
Calişkan et al., 1995 [6]	G	TUR	100	SC, MI	93.48	-	4.35	-	2.17	-	-	-	-
Sert & Bayirli, 2004 [14]	G	TUR	100	SC M	91.0	3.0	4.0	2.0	-	-	-	-	-
			100	F	96.0	-	-	4.0	-	-	-	-	-
Peiris & Roshan, 2008 [24]	G	LKA	87	SC	94.2	2.3	-	-	-	1.2	-	-	-
		JAP	83	SC	98.8	-	1.2	-	-	-	-	-	-
Weng et al., 2009 [15]	G	CHN	65	SC	75.4	20	1.5	3.1	-	-	-	-	-
Altunsoy et al., 2014 [8]	G	TUR	773	CBCT M	96.8	0.6	0.8	-	1.8	-	-	-	-
			750	CBCT F	98.7	0.1	0.1	0.7	0.4	-	-	-	-
Somalinga et al., 2014 [25]	G	IND	250	CBCT	81.6	2.8	11.6	0.8	2	-	-	-	1.2
Da Silva et al., 2016 [10]	G	BRA	200	CBCT	100.0	-	-	-	-	-	-	-	-
Martins et al., 2017 [26]	G	POR	962	CBCT	98.6	1.1	0.2	0.1	-	-	-	-	-
Jain P. et al., 2017 [12]	G	IND	100	CBCT	96.0	3.0	1.0	-	-	-	-	-	-
Plascencia et al., 2017 [27]	G	WE MEX	32	μ-CT	93.7	-	3.1	-	-	-	-	-	3.1
Martins et al., 2018 [28]	G	POR	368	CBCT M	99.5	0.5	-	-	-	-	-	-	-
			631	CBCT F	98.1	1.4	0.3	0.2	-	-	-	-	-
Martins et al., 2018 [29]	G	CHN	240	CBCT	100.0	-	-	-	-	-	-	-	-
		WE	999	CBCT	98.6	1.1	0.2	0.1	-	-	-	-	-
Razumova et al., 2018 [30]	F	RUS	540	CBCT	100.0	-	-	-	-	-	-	-	-
Pan et al., 2019 [31]	G	MYS	404	CBCT	100.0	-	-	-	-	-	-	-	-
Mashyakhy & Gambarini, 2019 [32]	G	SAU	184	CBCT M	97.8	-	2.2	-	-	-	-	-	-
			200	CBCT F	100.0	-	-	-	-	-	-	-	-
Nikkerdar et al., 2020 [33]	G	IRN	250	CBCT	95.6	-	2.0	-	2.4	-	-	-	-
Karobari et al., 2020 [13]	G	MAL	843	CBCT M	99.6	-	0.2	-	0.1	-	0.1	-	-
			849	CBCT F	99.6	-	0.1	-	0.2	-	0.1	-	-
Almohaimede et al., 2021 [7]	G	SAU	634	CBCT	97.94	0.47	0.47	-	1.1	-	-	-	-
Iqbal et al., 2022 [11]	G	SAU	343	CBCT M	98.2	-	1.8	-	-	-	-	-	-
			227	CBCT F	97.8	-	2.2	-	-	-	-	-	-
Buchanan, 2022 [9]	G	ZAF	393	CBCT	94.9	0.5	3.1	-	0.8	0.5	0.2	-	-

*Comp* Comparative study design, *Met* Research methodology, *Q/R* Quality rank, *PP* Country 3-digit code of population investigated, ^a^no classification given/possible, *SC* staining and clearing method, *Rx* Radiographic method, *Gr* Grinding method, *MI* Microscopic method, *CBCT* Cone beam computed tomography, *F* Female, *M* Male

## Discussion

This study aims to systematically review the literature regarding the internal morphology and root canal configuration (RCC) of maxillary canines (MxC) to provide the clinician with an overview that should lead to better understanding to make better treatment decisions leading to a better outcome in root canal treatments.

The present systematic review shows that the Briseño Marroquín et al.’s type 1–1-1/1 RCC of maxillary canines is the most frequently observed root canal configuration across all studies, with the lowest frequencies of this RCC type reported by Weng et al. [15] and Somalinga Amardeep et al. [25] at 75.4% and 81.6%, respectively. In half of the 22 studies analyzed, a Briseño Marroquín et al.’s 2–2-1/1 RCC (type II according to Vertucci and Weine) was reported as the second most common RCC with a frequency between 0.1 and 20% [12, 15, 24–26, 28, 29]. Nearly all studies examined reported an Briseño Marroquín et al.’s RCC of 1–2-1/1 (Vertucci's III, while the Weine et al. classification does not include this RCC type) as the third most common RCC with a frequency between 0.1 and 11.6% [7, 8, 12, 13, 26, 28, 29, 33]. In all studies, other Briseño Marroquín et al.’s RCCs such as 2–2-2/2, 1–1-2/2, 2–1-2/2, 1–2-1/2, and 1–1-3/3 (Vertucci types IV, V, VI, VII, VIII) were reported less frequently. However, one of the twenty-two included studies were published before the existence of Vertucci’s classification [5], reporting only a single root with RCC type 1–1-1/1 (Ve I).

There are plenty of research methods that have been used to examine the internal root canal morphology [1, 5, 6, 8, 27]. However, nowadays, micro-computed tomography (micro-CT) imaging is widely accepted as the gold standard for ex vivo research for internal tooth morphology and root canal configuration [18]. Advances in non-destructive digital three-dimensional imaging systems such as cone beam computed tomography (CBCT) and micro-CT imaging can provide data that simplify the analytical process for describing internal morphology [34]. These methods offer the possibility of obtaining both quantitative and qualitative information about the samples noninvasively and without destroying them, and of reusing the samples for future examinations, if necessary, in contrast to alternative techniques that were frequently used in the past, such as staining and clearing [35, 36]. Although CBCT enables less detailed visualization of fine structures than micro-CT, it allows clinical use in vivo, which is currently not possible with either staining and clearing techniques or with humans using micro-CT due to the high radiation exposure.

Various classification systems for root canal configuration are given in the literature [1–4], whereby the systems of Vertucci [1] and Weine et al. [2] were the most used systems for many years. In the meantime, more modern systems are used to describe the root canal configuration, which allows additional information about the entire root [3, 4].

Six of the twenty-two included studies compared sex differences [8, 11, 13, 14, 28, 32], whereby all studies were examined by CBCT, except for one study using the staining and clearing method [14]. All authors reported that Briseño Marroquín et al.’s RCC of 1–1-1/1 was most observed in both sexes, with up to 99.5% in men and up to 100% in women. It was also observed that there were sex-specific differences in the comparison of studies from Europe and Asia, resulting in increased frequencies regarding different root canal configurations. Furthermore, differences in the studies can be explained by the research methodologies used or ethnic origin. While sex differences were still documented in various studies, the age of the subjects or patients was reported in some studies, but not included in the analysis of the respective studies.

Although the most common Briseño Marroquín et al.’s RCC for MxC is 1–1-1/1 (Ve I), the clinician should always be aware of the complex internal root canal morphology in up to 25% of cases. These could include connecting canals or even accessory root canals that cannot be prepared mechanically, emphasizing the importance of chemical root canal irrigation. The application of an adequate irrigation protocol and a careful obturation technique therefore has an important impact on reducing complications or errors that could compromise the outcome of root canal treatment.

Various limitations should be mentioned, such as possible distortions in the selected studies due to the methodology used or possible artifacts, especially in the digital imaging of CBCT and micro-CT, but also limitations of the selected subjective evaluation criteria and description methodology for root canal configuration. Although it is known that aging, caries or even tooth wear can cause a narrowing of the root canal system due to secondary dentin deposits [24], it has also been reported that they have only a minimal effect on the morphology of the main root canal [24, 37]. Unfortunately, some studies explicitly stated that no information on sex and age was available [25, 27]. Although the age of the patients examined by CBCT may have been available in several included studies, this was unfortunately not included in the analysis regarding the change in root canal configuration. A comparison with ethnic groups of similar mean age would be interesting, with few studies in molars and none for maxillary canines suggesting that age may influence the configuration of the root canal system in certain tooth types [29, 38] or that the frequency of complex root canal configurations as well as the presence of second mesial root canals in molars may decrease with age [39].

## Conclusions

Within the limitations of the current systematic review and meta-analysis, the following conclusions can be drawn:

- The most frequently observed RCC of MxC is the 1–1-1/1 (Vertucci’s and Weine’s et al. type I), followed by a 2–2-1/1 (Vertucci’s and Weine’s et al. type II) and 1–2-1/1 (Vertucci’s type III).
- 25% of cases harbor the possibility of a more complicated RCC, which should always be taken into consideration by the clinician.
- The most frequently used method for in vivo research on the root canal morphology of MxC, nowadays, is CBCT.
- A significantly higher number of RCC of 2–2-1/1 (Ve II), 1–2-1/1 (Ve III), and 1–1-2/2 (Ve V) were observed in males and 2–2-2/2 (Ve IV) in females. No sex differences in the RCCs of 1–1-1/1 (Ve I) and 1–2-1/2 (Ve VII) were observed.

### Supplementary Information

Supplementary Material 1

Supplementary Material 2

## Data Availability

The authors confirm that the data supporting the findings of this study are available within the article and its supplementary materials.
